# Paramedic clinical decision making during high acuity emergency calls: design and methodology of a Delphi study

**DOI:** 10.1186/1471-227X-9-17

**Published:** 2009-09-21

**Authors:** Jan L Jensen, Pat Croskerry, Andrew H Travers

**Affiliations:** 1 Division of Emergency Medical Services, Department of Emergency Medicine, Dalhousie University, 1796 Summer Street, Room 3021, Halifax, Nova Scotia, Canada; 2 Emergency Health Services Nova Scotia, Dartmouth, Nova Scotia, Canada; 3 Atlantic Regional Training Centre, Masters in Applied Health Services Research Program, Dalhousie University, Halifax, Nova Scotia, Canada

## Abstract

**Background:**

The scope of practice of paramedics in Canada has steadily evolved to include increasingly complex interventions in the prehospital setting, which likely have repercussions on clinical outcome and patient safety. Clinical decision making has been evaluated in several health professions, but there is a paucity of work in this area on paramedics. This study will utilize the Delphi technique to establish consensus on the most important instances of paramedic clinical decision making during high acuity emergency calls, as they relate to clinical outcome and patient safety.

**Methods and design:**

Participants in this multi-round survey study will be paramedic leaders and emergency medical services medical directors/physicians from across Canada. In the first round, participants will identify instances of clinical decision making they feel are important for patient outcome and safety. On the second round, the panel will rank each instance of clinical decision making in terms of its importance. On the third and potentially fourth round, participants will have the opportunity to revise the ranking they assigned to each instance of clinical decision making. Consensus will be considered achieved for the most important instances if 80% of the panel ranks it as important or extremely important. The most important instances of clinical decision making will be plotted on a process analysis map.

**Discussion:**

The process analysis map that results from this Delphi study will enable the gaps in research, knowledge and practice to be identified.

## Background

### Clinical Decision Making

Clinical Decision Making (CDM) (also known as clinical reasoning, clinical judgment) has been defined and studied in medicine over the last few decades [1]. Other health professions have also investigated how practitioners made decisions, such as nursing [2,3]. However, to date, very little research on CDM has been conducted in the paramedic population. Presumably, weak abilities in CDM lead to clinical errors, which are prevalent in healthcare [4] and are often the causes of lapses in patient safety. Therefore, CDM is an essential component of the body of research on patient safety, as it relates to emergency medical services (EMS).

The care that patients receive in the out-of-hospital setting likely has important repercussions on clinical outcome and patient safety. Patient assessment and treatment can vary substantially, from simple ambulance runs to calls that require expedient decision making and action by paramedical personnel. There are many factors that can influence outcome, including the acuity of the patient's injury or illness, the location of the patient, the wants and needs of the patient and their family, the resources available to the paramedics, the level of care provided by practitioners, and the number, complexity and time dependence of interventions required, both on scene and *en route *to the hospital. As the scope of practice of paramedics continues to expand and the sophistication of EMS systems evolves, it is essential to evaluate and expand the current state of knowledge on paramedic CDM.

### Paramedics and EMS in Canada

In Canada, there are three recognized levels of paramedics: Primary Care Paramedics (PCP), Advanced Care Paramedics (ACP), and Critical Care Paramedics (CCP) [5]. The ACP scope of practice has traditionally included advanced airway management, intravenous (IV) access, IV drug administration, and other skills [5]. Across Canada, recent changes have seen ACPs provide additional interventions, such as 12-lead electrocardiogram interpretation, administration of thrombolytics for acute myocardial infarction and application of continuous positive airway pressure ventilation for acute shortness of breath [6,7].

There is a paucity of literature related to EMS patient safety and paramedic CDM. Some work has been done on errors on specific clinical interventions, such as endotracheal intubation [8,9], and on error reporting patterns of paramedics [10]. Isolated reports have been found on paramedics' decisions to initiate specific interventions, such as IV lines [11] and rapid sequence induction for intubation [12]. Given the expanding role of paramedics, this area would assume increasing importance.

### The Delphi Technique

Delphi studies are frequently used in healthcare, with the goal of establishing consensus on a particular topic [13]. Iterative rounds of structured surveys are administered to a group of experts on the topic, who rank each item. On subsequent rounds, each panel member views the ranking they assigned to each item, as well as the group mean ranking. Participants have the opportunity to revise their ranking, taking into consideration the group mean. The rounds continue until consensus is achieved, or a predetermined end point is met. The technique is beneficial because consensus can occur in an anonymous format, without physically bringing experts together. Four key features make Delphi studies well suited for determining group consensus: anonymity of responses; iteration with controlled feedback; statistical group response; and, the use of experts [14]. The results of a Delphi study can help direct future research, continuing education and allocation of resources. The obvious limitation of such a consensus study is the results are not linked to actual patient outcomes, and therefore the results are only as good as the panel members' opinions. Nevertheless, the opinion and experiences of EMS experts is useful to inform the most important instances of CDM that occur during a high acuity ambulance calls. The CDM instances that are found to be the most important will be organized in a process analysis map. This strategy has been developed for emergency medicine using a modified Delphi approach [15-17]. The model will enable gaps in research, knowledge and practice to be identified.

### Objective

Using expert consensus, the instances of clinical decision making that are required by paramedics on typical high acuity ambulance calls will be determined, in terms of their importance to clinical outcome and patient safety.

## Methods and design

### Study Design

This cross-sectional study will use the Delphi technique to achieve consensus amongst EMS experts on the most important instances of clinical decision making by paramedics during high acuity emergency calls, in the ground ambulance setting. These instances will be scored on importance, based on their anticipated impact on patient clinical outcome and patient safety. The final consensus will be used to develop a process analysis map of paramedic clinical decision making.

### Setting and Population

Subjects for this study will be recruited using purposive and criterion sampling. The goal is to have a sample of EMS experts from across Canada, which will include EMS medical directors and paramedic leaders. Two key organizations will be targeted for recruitment: the Canadian Association of Emergency Physicians EMS Committee and the EMS Chiefs of Canada. An expression of interest posting will be distributed throughout these two organizations. Recipients of the posting will be invited to distribute it to paramedics or EMS medical directors who fit our definition of 'expert', and are likely to be interested and willing to participate. Those interested will be invited to email one of the investigators.

Delphi studies recruit experts to give their opinion on a particular subject, with the goal of achieving consensus amongst the group [18]. Experts will be considered paramedics or medical directors with greater than eight years of experience. Paramedic experts may presently work primarily in a clinical out-of-hospital setting (ground or air ambulance), or primarily in a quality and learning/quality assurance division, and must be of the ACP level or higher. This latter requirement was established to ensure external validity for all levels of paramedics. As the vast majority of ACPs were PCP prior to their ACP training, they can incorporate this perspective in their responses, and it is assumed ACPs would be capable of more complex clinical decisions, given their broader scope of practice. EMS medical directors must currently oversee a paramedic service, and be actively involved in providing clinical quality assurance feedback to paramedics on their clinical performance.

The choice of participants in a Delphi study is essential to its success, and the validity of the results [19]. The investigators will select participants from those who email their interest to participate. Participants will be anonymously described in dissemination of the results, so readers can have an awareness of the panel composition. In keeping with the typical sample size for Delphi studies, 15 - 20 participants will be recruited for this study.

This study has received approval from the Capital District Health Authority REB (Halifax, Nova Scotia): CDHA-RS/2009-372. All participants provided written informed consent via fax to our office in Halifax.

### Method of Measurement

Participants will be emailed a link to an online survey site [20] for anonymous responding - a key aspect of the Delphi method. This is especially important in this panel, which will be a mix of paramedics and medical directors. Anonymous responses will help to ensure that participants are responding according to their own thoughts and beliefs, and not because they are influenced by opinion leaders on the panel [13]. The responses will not be anonymous to the investigators, however, but will be kept confidential.

The first round of the Delphi study will be open for two weeks. Participants will enter any instances of paramedic CDM that they feel are important during a high acuity ambulance call in a free text box. An additional text box will be provided for respondents to enter any further thoughts or elaborations. The responses will be analyzed and categorized, maintaining the original wording of the respondent as much as possible [14].

The second round of the survey will be sent back out for the panel to review, and will also be open for two weeks. Participants will score each instance on a Likert scale, in terms of its importance to patient clinical outcome and safety. They will be given the opportunity to add new CDM instances, and provide additional free-text comments.

On the third round, the mean rankings for each instance of CDM and the respondents own response will be available for the individual participants' review (i.e., each participant will see their own responses, and all will see the group mean responses). As the investigators will be returning each respondents scoring on each item from the previous round to them, along with the mean score from the group, the responses cannot be anonymous to the investigators. On the third round and possibly next round, participants can revise their ranking for any of the CDM instances, based on viewing the group mean and their own score. The survey will be re-sent until this consensus is achieved, to a maximum of four rounds. This limit will be instituted to avoid sample fatigue.

### Data Analysis

It is essential to define the meaning of 'consensus' *a priori *[19]. For this study, consensus for each CDM item will be set at 80% or more of respondents grading it as 4 (Important - in most instances these decisions will impact patient clinical outcome or patient safety), or, 5 (Extremely important -very likely to impact patient clinical outcome or patient safety). Once an item has reached this level of consensus, it will be removed from the list and not appear for re-ranking in subsequent rounds. Data will be entered into the statistical software program SPSS. Agreement will be measured between the paramedic and medical director respondents using concordance statistics (kappa scores) and t-tests. Response rate for each round will be reported, as well as descriptive statistics of the panel demographics.

The free text additional comments from each round will be analyzed using qualitative analysis software after the final round. The findings of the thematic analysis of the free text will be used to give context to the Delphi findings.

The instances of paramedic CDM that are found to be important to clinical outcome and patient safety will be plotted onto a process analysis map. This map will be sent to the panel members for comment at the end of the study.

### Pilot Study

A pilot study has been conducted. Three paramedics and two emergency physicians, one of whom is a study investigator (AT) completed three rounds. The online surveys were edited based on pilot participant feedback. No results from the pilot will be used in the actual study.

## Discussion

This study will provide insight into the most important clinical decisions paramedics make during high acuity emergency calls. The implications for such knowledge include exposing research and education gaps, establishing priorities for paramedic practice, and providing direction for professional development and patient safety initiatives in the EMS setting.

## Abbreviations

CDM: clinical decision making; EMS: emergency medical services; PCP: primary care paramedic; ACP: advanced care paramedic; CCP: critical care paramedic; IV: intravenous.

## Competing interests

The authors declare that they have no competing interests.

## Authors' contributions

JLJ conceived the study and secured funding. PC assisted with design and intellectual background. AT also assisted with study design and refining the study after the pilot. PC and AT revised the manuscript critically for important intellectual content. All authors read and approved the final manuscript.

## Pre-publication history

The pre-publication history for this paper can be accessed here:


